# What Are the Ethical Issues Surrounding Extended Reality in Mental Health? A Scoping Review of the Different Perspectives

**DOI:** 10.3390/bs15101431

**Published:** 2025-10-21

**Authors:** Marie-Hélène Goulet, Laura Dellazizzo, Simon Goyer, Stéphanie Dollé, Alexandre Hudon, Kingsada Phraxayavong, Marie Désilets, Alexandre Dumais

**Affiliations:** 1 Faculty of Nursing, Université de Montréal, Montreal, QC H3T 1A8, Canada; marie-helene.goulet@umontreal.ca (M.-H.G.);; 2 Research Center of the Institut Universitaire en Santé Mentale de Montréal, Montreal, QC H1N 3M5, Canada; laura.dellazizzo@umontreal.ca (L.D.); alexandre.hudon.1@umontreal.ca (A.H.);; 3 Institut National de Psychiatrie Légale Philippe-Pinel, Montreal, QC H1C 1H1, Canada; 4 Research Center CIUSSS-NIM, Hôpital du Sacré-Cœur de Montréal, Montreal, QC H4J 1C5, Canada; 5 Faculty of Medicine, Departement of Psychiatry and Addictology, Université de Montréal, Montreal, QC H3T 1J4, Canada; 6 Institut Universitaire en Santé Mentale de Montréal, Montreal, QC H1N 3M5, Canada

**Keywords:** extended reality, virtual reality, virtual environments, virtual agents, scoping review, mental health, psychiatry, ethical issues

## Abstract

As extended reality (XR) technologies such as virtual and augmented reality rapidly enter mental health care, ethical considerations lag behind and require urgent attention to safeguard patient safety, uphold research integrity, and guide clinical practice. This scoping review aims to map the current understanding regarding the main ethical issues arising on the use of XR in clinical psychiatry. Methods: Searches were conducted in 5 databases and included 29 studies. Relevant excerpts discussing ethical issues were documented and then categorized. Results: The analysis led to the identification of 5 core ethical challenges: (i) Balancing beneficence and non-maleficence as a question of patient safety, (ii) Altering autonomy by altering reality and information, (iii) data privacy risks and confidentiality concerns, (iv) clinical liability and regulation, and v) fostering inclusiveness and equity in XR development. Most authors have stated ethical concerns primarily for the first two topics, whereas the remaining four themes were not consistently addressed across all papers. Conclusions: There remains a great research void regarding such an important topic due the limited number of empirical studies, the lack of involvement of those living with a mental health issue in the development of these XR-based technologies, and the lack of clear clinical and ethical guidelines regarding their use. Identifying broader ethical implications of such novel technology is crucial for best mental healthcare practices.

## 1. Introduction

Mental health systems face rising demand while resources and workforce capacity remain constrained, creating persistent gaps in access and quality. Against this backdrop, and specifically within mental healthcare, there is growing interest in extended reality (XR) as a set of tools that could expand capacity and personalize care. XR is an umbrella term spanning the reality–virtuality continuum—from full immersive virtual reality (VR) to augmented reality (AR), which overlays digital content onto the physical world (Milgram & Kishino, 1994). VR typically uses head-mounted displays to deliver a fully computer-generated environment, often leveraging multisensory cues to enhance immersion and presence (Slater, 2009; Cipresso et al., 2018; Park et al., 2019; Torous et al., 2021). AR is commonly defined—following a widely cited, consensus formulation—as combining real and virtual content, being interactive in real time, and being registered in three dimensions (Azuma, 1997; Billinghurst et al., 2015). In psychiatric applications, some XR environments also include avatars. These are computer-generated representations of either humans or other characters and are commonly guided by a professional (Rudschies & Schneider, 2024). Additionally, virtual embodied conversational agents are computer systems usually based on artificial intelligence (AI) with the aim to simulate human conversation while interacting with users in an autonomous manner (Rudschies & Schneider, 2024).

Among the various areas of healthcare where XR can make a significant impact, mental health stands out, having seen a wide range of developments over the past decade (Cieślik et al., 2020; Freeman et al., 2017; Wiebe et al., 2022). Psychiatric disorders remain one of the top significant public health challenges and are among the top leading cause of disability and overall disease burden worldwide (World Health Organization (WHO), 2022). XR has emerged as an effective and promising tool for assessing a large range of mental disorders and to expand the range of psychotherapy modalities (Carl et al., 2019; Dellazizzo et al., 2020; Freeman et al., 2017; Maples-Keller et al., 2017; Rus-Calafell et al., 2018; Valmaggia et al., 2016). Notably, a recent systematic review by Wiebe et al. (2022) included 721 studies on the use of VR in mental health, and highlighted that such technology can benefit several disorders, including anxiety disorders, autism spectrum disorder, posttraumatic stress disorder, dementia, schizophrenia spectrum disorders, and addiction disorders.

To date, the most supported application is VR exposure therapy for anxiety and trauma-related disorders, with numerous meta-analyses showing its efficacy (Carl et al., 2019). XR also provides new possibilities to go beyond current treatment techniques for complex and challenging patients. For instance, a few studies have explored the usability of VR in the context of forensic mental health and appears as a suitable tool for the prevention of aggression (Fromberger et al., 2018; Klein Tuente et al., 2020; Sygel & Wallinius, 2021). In the last few years, avatars have been increasingly used for treating persecutory auditory verbal hallucinations in treatment-resistant schizophrenia patients. Findings have shown positive effects on these difficult to treat patients (Craig et al., 2018; Dellazizzo et al., 2021).

There are several advantages of XR for training, diagnostic and therapeutic purposes, including reduced cost, interactivity, and safety (Li et al., 2017). XR therefore entails several advantages for mental healthcare, such as real-time performance and behavioral monitoring, personalised exposure and creation of real-world situations that may otherwise be impossible (Best et al., 2022). Moreover, virtual environments have been shown to produce physiological changes consistent with emotional responses to real-world scenarios and can elicit different symptoms (i.e., paranoia, cravings, anxiety) (Costanzo et al., 2014; Freeman et al., 2003; Kuntze et al., 2001; Lee et al., 2003; Owens & Beidel, 2015). Since virtual environments are computer-generated, patients can experience these environments in a safe and controlled manner in a clinical setting (Bell et al., 2020). XR may offer users experiences involving scenarios that would be too difficult or too dangerous to practice real world (Baus & Bouchard, 2014) and offer the illusion of being located inside the rendered virtual environment (Baus & Bouchard, 2014; Slater, 2009). XR experiences may consequently meet patients’ needs, abilities, or preferences (Bell et al., 2020). Recent work further documents effectiveness and implementation in mental-health contexts (e.g., randomized trials of VR relaxation; implementation processes; avatar-based digital therapies) (Veling et al., 2021; M. M. T. E. Kouijzer et al., 2023; Garety et al., 2024). Beyond VR, other XR modalities have also been explored: AR as an aid to cognitive behavioral therapy for anxiety disorders and mixed reality environments for adaptive mental-health support, with growing evidence syntheses in specific conditions such as obsessive–compulsive disorder (Rajkumar, 2024; Navas-Medrano et al., 2024; Colman et al., 2024).

Besides the above-mentioned potential of these technologies, there are limiting factors to the implementation of XR in clinical practice beyond research settings, comprising the lack of high-quality evidence XR programs, the absence of training and standardized evidence-based packages, fears that technology may deter patient engagement and lack of infrastructure to support the technology within clinical settings (Bell et al., 2020; Selaskowski et al., 2024).

Furthermore, it has been demonstrated that digital applications are often designed without any specific ethical considerations (Fiske et al., 2019; Ienca et al., 2018). These challenges may go beyond the training received by professionals and directly affect patient care—for example, ensuring safe exposure, informed consent, and respect for autonomy when using immersive systems—while also shaping research integrity (transparent reporting, protection of participants, and governance of data) and clinical practice (clear protocols, competency requirements, and accountability) (Rizzo & Koenig, 2017; Chung et al., 2021). As such, clinicians may be unprepared for legal and ethical challenges (e.g., privacy, electronic security, accountability) involved in using virtual environments (Parsons, 2021). Moreover, ethical issues may arise depending on the type of population being assessed or treated. For example, the distortion of realities with XR may have unjustified consequences on how users relate to the real world, which may be especially challenging for patients with pre-existing reality distortion (Bell et al., 2020). Utilizing XR on patients with dementia or in forensic settings may also raise ethical intricacies, particularly regarding autonomy and vulnerability, requiring proportionate safeguards and context-specific oversight (Ligthart et al., 2022).

Ethical implications consequently require further probing to identify pertinent concerns surrounding key principles in biomedical ethics, such as autonomy, beneficence, non-maleficence and justice as well as to anticipate concerns (T. Beauchamp & Childress, 2019; T. L. Beauchamp, 2007). This theoretical framework describes the following ethical principles: (i) Autonomy, which includes informed consent and the right of the patients to choose or refuse treatment; (ii) beneficence, which means that the professional acts in the best interest of the patient; (iii) non-maleficence, which entails minimizing risks; and (iv) justice, which concerns the fair distribution of benefits, burdens, and health-care resources.

Given the rapid development of XR possibilities, several key stakeholders have questioned the ethical dilemmas in various types of literature. It has thus been noted that each technological modality brings its own nuanced ethical challenges (Bond et al., 2023; D’Alfonso et al., 2019; Kellmeyer, 2018; Kellmeyer et al., 2019; Ligthart et al., 2022; Marloth et al., 2020; Parsons, 2021; Slater et al., 2020; Torous & Roberts, 2017; Wykes et al., 2019). A scoping review is necessary to assess the breadth of existing knowledge on the topic, to synthesize the literature, and to highlight key findings while facilitating a dialogue between them.

In this paper, we aim to provide an in-depth analysis of the main ethical issues arising from mental health literature on the use of XR in clinical settings. Identifying the ethical implications of such novel technologies is crucial for exploring research avenues that will advance these ethical considerations and progressively help shape ethical guidelines to regulate these practices in mental healthcare.

## 2. Materials and Methods

This scoping review is based on the methodological framework of the Joanna Briggs Institute (JBI) (Peters et al., 2020). This approach aims to provide an overview of the existing literature on a specific topic and identify gaps in knowledge to guide future research. This manuscript follows the Preferred Reporting Items for Systematic Reviews and Meta-Analyses extension for Scoping Reviews (PRISMA-ScR) checklist (see Supplementary Material S1). The review process was structured around five key steps: (a) defining the research question (what are the main ethical issues arising from mental health literature on the use of XR in clinical settings); (b) search strategy; (c) study selection; (d) data extraction; and (e) data analysis and synthesis (Peters et al., 2020). Two reviewers conducted all phases—title/abstract screening, full-text eligibility, data extraction, and analysis—and held regular consensus meetings with the research team.

### 2.1. Search Strategy and Eligibility Criteria

The inclusion and exclusion criteria established to meet the study objective follow the PCC framework: Population, Concept, and Context (see Table 1).

The online databases of PubMed, Medline, EMBASE, PsycINFO, and Google Scholar were systematically searched by our team with the help of a librarian specialized in mental health to identify all relevant research reporting ethical issues associated with the use of XR in mental health. Searches used mesh terms and keywords in the title as well as abstract that were inclusive for ethical issues as defined by the biomedical ethics theoretical framework (T. Beauchamp & Childress, 2019) or identified as an ethical issue by the authors themselves, XR, and mental health (e.g., mental disorders, psychiatry, mental health). The search syntax was tailored for each database (see Supplementary Material S2 an example). Searches were limited to English and French language sources. No setting, date or geographical restrictions were applied. The search was last updated in January 2025. We included all study designs including theoretical and empirical research (qualitative, quantitative, mixed), as well as literature reviews and commentaries. Papers were considered for inclusion if they specifically addressed XR environments. Text-based agents, such as chatbots, were excluded because the goal was to include what contains a virtual environment. Studies focusing solely on virtual healthcare, telemedicine, or artificial intelligence without involving a virtual environment were also excluded. We also excluded articles dealing with educational purposes or training professionals. References were exported to the EndNote version 21 software. Additional records were identified through cross-referencing. To ensure consensus, discussions on the inclusion of articles were held regularly with team members.

### 2.2. Study Selection

**Figure 1:**
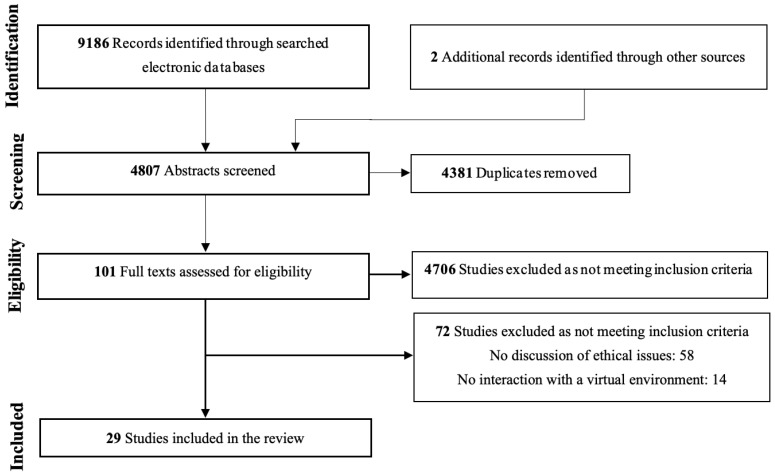
PRISMA flow-chart.

After removing duplicates, the identified articles were independently screened by LD and SG based on their titles and abstracts. A pilot test was conducted on 25 articles, as suggested by Peters et al. (2020), to compare their selection. An in-depth review of full-text articles included in the scoping review was then performed to assess their eligibility based on the inclusion and exclusion criteria. In cases of disagreement regarding article selection, a third evaluator has been consulted. The reasons for article exclusion have been documented and the results of the selection process are presented in a PRISMA-ScR flow diagram (Figure 1).

### 2.3. Data Extraction

The information was recorded in a data charting format, with the components of the extraction form identified through team discussions and inspired by the extraction grid proposed by the JBI, which was adapted to align with the aim of this scoping review. Key information included authors; year of publication; country of origin; type of study; aim; theoretical framework or perspective; methodology; population; type of XR platform; healthcare setting; sample size; and identified knowledge gaps related to the topic of interests. To facilitate close, text-proximal analysis by team members who had not read the full texts, ethics-related excerpts were copied verbatim into the chart, with citation details to ensure traceability.

### 2.4. Data Analysis and Synthesis

We employed an inductive-deductive approach inspired by the qualitative content analysis method (Miles et al., 2018). All relevant excerpts discussing ethical issues were documented and categorized by the authors into ethical spheres aligned with the principles of biomedical ethics (T. Beauchamp & Childress, 2019; T. L. Beauchamp, 2007), while remaining open to emerging concepts. Patterns and contradictions among the studies were identified. Research synthesis was conducted based on the various subcategories grounded in this framework. Frequent meetings involving the authors of this review were held to refine the themes. A summary of the findings was then drafted and discussed with all authors to develop a thematic mapping that reached consensus.

## 3. Results

This section first presents the characteristics of the included articles, followed by the main themes emerging from the analysis.

### 3.1. Characteristics of Included Articles

The search on the databases led to 9186 studies. Additional records were identified through other sources (*n* = 2). Once removing duplicates and irrelevant studies by screening articles based on their titles and abstracts, 98 full texts were read leading to the inclusion of 29 studies (see Table 2). The reviewed articles included opinions or commentary pieces (*n* = 11), narrative or descriptive reviews (*n* = 11), qualitative studies (*n* = 5), and quantitative studies (*n* = 2). These studies were picked up for data analysis and research synthesis. Table 2 presents the characteristics and methods of the assessed articles.

### 3.2. Themes Associated with Ethical Issues Arising on the Use of Extended Reality in Clinical Psychiatry

- Theme 1. Balancing beneficence and non-maleficence as a question of safety

Challenges in balancing beneficence and non-maleficence were identified in 19 articles and cluster into four subthemes: (1) physical adverse effects; (2) psychological and emotional unintended consequences; (3) population- and context-specific risks; and (4) clinical issues (Anderson et al., 2004; Bell et al., 2020; Botella et al., 2009; Chung et al., 2023; Chung et al., 2021; Cornet & Van Gelder, 2020; Fromberger et al., 2018; Georgieva & Georgiev, 2019; Kellmeyer, 2018; Klein Haneveld et al., 2023; M. T. E. Kouijzer et al., 2024; Kruk et al., 2019; Ligthart et al., 2022; Marloth et al., 2020; Ozerol & Andic, 2023; Parsons, 2021; Pellicano, 2023; Selaskowski et al., 2024; Washburn et al., 2021).

*Physical adverse effects.* Mental health professionals and patients should be alert to the potential physical adverse effects related to XR environments, with simulator sickness and XR aftereffects being the most discussed by authors (Anderson et al., 2004; Bell et al., 2020; Botella et al., 2009; Chung et al., 2023; Chung et al., 2021; Fromberger et al., 2018; Georgieva & Georgiev, 2019; Kellmeyer, 2018; Klein Haneveld et al., 2023; Parsons, 2021). As may be observed in motion sickness, several authors discuss how a minority of patients may have a greater sensitivity to being immersed in XR environments and may experience, during or post-immersion, varying degrees of manifestations, including fatigue, headache, eye strain, nausea, disorientation, ataxia, and vertigo, disturbed locomotion, perceptual-motor disturbances, flashbacks and lowered arousal (Anderson et al., 2004; Bell et al., 2020; Botella et al., 2009; Kellmeyer, 2018; Kruk et al., 2019; Parsons, 2021; Selaskowski et al., 2024).

*Psychological and emotional unintended consequences.* Other relevant considerations discussed by authors with respect to the use of XR have been related to the psychological and emotional side effects of specific XR experiences, including the addictive potential of VR technology (Georgieva & Georgiev, 2019; Marloth et al., 2020), reality distortion (Bell et al., 2020; Georgieva & Georgiev, 2019; Marloth et al., 2020), depersonalization or derealization (Kruk et al., 2019; Parsons, 2021; Selaskowski et al., 2024), trigger symptoms and distress (ex., traumatization, re-traumatization, hallucinations) (M. T. E. Kouijzer et al., 2024; Marloth et al., 2020; Ozerol & Andic, 2023; Pellicano, 2023). Conversely, not all XR-related emotional effects are adverse: in a randomized crossover trial of 50 outpatients with anxiety, psychotic, depressive, or bipolar disorders, VR-based relaxation produced a significantly greater reduction in total negative affective state compared with standard relaxation (Veling et al., 2021), suggesting potential short-term emotional benefits when appropriately designed and supervised.

*Population- and context-specific risks.* Some have questioned whether XR would benefit those with more severe psychopathologies (e.g., psychosis) or interact with psychotropic medications (Botella et al., 2009; Chung et al., 2021; Parsons, 2021). Additionally, the use of VR technology appears to present distinct risks when applied to forensic populations (Cornet & Van Gelder, 2020; Fromberger et al., 2018; M. T. E. Kouijzer et al., 2024; Ligthart et al., 2022). For instance, for child abusers, exposure to virtual children as a treatment modality could theoretically induce sexual arousal even after the XR experience is over; virtual risk situations could also be misused as child pornography (Cornet & Van Gelder, 2020). Mental health professionals must consider whether they are acting in the best interest of patients (beneficence) and consider the risks of immersion (non-maleficence) (Parsons, 2021).

*Clinical issues.* Qualitative studies indicates variable clinician perceptions of risk—some see no added risk beyond routine practice, whereas others report concerns (e.g., simulation sickness, distress, dissociation, panic attacks) (Chung et al., 2023; Chung et al., 2021). Additional concerns include maintaining appropriate boundaries around physical touch to prevent patient injury while in a XR environment (risk of misinterpretation (Chung et al., 2021), ensuring fitness to perform tasks post-session (e.g., driving) given potential aftereffects (Anderson et al., 2004), managing session dosage (Botella et al., 2009; Kellmeyer, 2018), and allowing sufficient time to re-acclimate to the non-VR world (Washburn et al., 2021).

- Theme 2. Altering autonomy by altering reality and information

We distinguish three subthemes that connect simulated realities to autonomy and informed consent: (1) deception and transparency; (2) vulnerabilities and reality-testing; and (3) informed consent as the operationalization of autonomy (Anderson et al., 2004; Campaner & Costerbosa, 2023; Chivilgina et al., 2021; Georgieva & Georgiev, 2019; Kellmeyer, 2018; Kellmeyer et al., 2019; Kruk et al., 2019; Ligthart et al., 2022; Luxton & Hudlicka, 2021; Marcoux et al., 2021; Marloth et al., 2020; Selaskowski et al., 2024; Whalley, 1995). Similarly, the issues concerning informed consent and patient autonomy were raised by 9 articles (Campaner & Costerbosa, 2023; Fromberger et al., 2018; Kellmeyer et al., 2019; Klein Haneveld et al., 2023; Ligthart et al., 2022; Marcoux et al., 2021; Marloth et al., 2020; Parsons, 2021; Whalley, 1995).

*Deception and transparency deception*. Authors explained how XR may create altering and prefabricated realities causing deception and lies to patients, while altering therapeutic relationships (Anderson et al., 2004; Campaner & Costerbosa, 2023; Chivilgina et al., 2021; Georgieva & Georgiev, 2019; Kellmeyer et al., 2019; Kruk et al., 2019; Ligthart et al., 2022; Marloth et al., 2020; Selaskowski et al., 2024; Washburn et al., 2021; Whalley, 1995). Deception notably occurs when patients may not know whether they are interacting with a human controlling the environment or whether it is controlled autonomously (Luxton & Hudlicka, 2021). In such a case, the patient’s autonomy would therefore not be respected. Some highlight that these realities might be more appealing to some patients leading to ethical concerns regarding the fact that it may provide an escape from reality leading to addiction and social withdrawal (Georgieva & Georgiev, 2019; Selaskowski et al., 2024; Whalley, 1995). Modern technologies, such as those using artificial agents, may likewise have human-like appearances and show empathy that may bring on concern about artificial relationships, whereas patients may develop feelings and attachment towards the simulated agent (Luxton & Hudlicka, 2021). Though it is worth noting that these immersive realities nonetheless have an unspecified loss of authenticity and result in a loss of certain aspects of social interaction, which may negatively affect patients (Campaner & Costerbosa, 2023; Kruk et al., 2019; Marloth et al., 2020). Some argue that patients should not be imposed in such environments with constraints that lack meaning and authenticity, which may even manipulate them into behavioral changes unconsciously, which affect a patients’ autonomy (Campaner & Costerbosa, 2023; Ligthart et al., 2022; Marloth et al., 2020). Moreover, it remains questionable whether patients could be themselves when interacting in virtual environments or whether sanctions should occur, for instance, in the case of violence against virtual characters (Chivilgina et al., 2021).

*Vulnerabilities and reality-testing.* On the other hand, some patients with reduced reality testing capacity, such as those with cognitive impairments or those with psychosis, may not distinguish “real” reality from the vivid and compelling details provided by the immersive reality, thereby deceiving the patient and potentially destabilizing them (Chivilgina et al., 2021; Kellmeyer et al., 2019; Luxton & Hudlicka, 2021; Marloth et al., 2020; Washburn et al., 2021). Individuals with disturbed self-perceptions, such as those experiencing eating disorders, may also face impacts on their identity and self-perception, potentially leading to feelings of loss of control (Chivilgina et al., 2021; Kellmeyer, 2018). They state that a certain amount of deception remains in such a therapy. It is moreover important to pay attention to the content of dialogue and limits to pressure, solicitation and suggestions by ensuring deontological guidelines are followed (Campaner & Costerbosa, 2023).

*Informed consent as the operationalization of autonomy.* Authors discuss the importance that patients must be informed, in a language that they understand, about the nature of the therapy (e.g., XR environment, type of exposure, duration of exposure), limitations of the therapy as well as the possibility of withdrawing from virtual exposure at any moment and any potential unpleasant effects or risks of XR immersion, which may persist in time (Fromberger et al., 2018; Klein Haneveld et al., 2023; Marcoux et al., 2021; Marloth et al., 2020; Parsons, 2021). Concerning the latter, patients should nonetheless be informed that the level of anxiety may increase at the early stages of immersion, though cumulative exposure is aimed at augmenting their tolerance, thereby improving their autonomy (Parsons, 2021). In the case patients are in contact with virtual characters (i.e., avatars), they must clearly understand the degree of agency of these characters to reduce the risks of strong attachments and adjust their expectations (Marcoux et al., 2021). They must also be informed if it is planned that the data that will be collected during the therapy (e.g., biomarkers) and any data that will be conserved in time, for instance, to develop algorithms and how data will be conserved (e.g., anonymized) (Marcoux et al., 2021). Informed consent should be a collaborative effort between the clinicians and patients, which may increase treatment efficacy, cooperation and trust (Parsons, 2021). Special attention must be paid to patients who are asked to consent and who have cognitive deficits (e.g., dementia) or who have a court order (e.g., offenders with mental disorders) (Fromberger et al., 2018; Kellmeyer et al., 2019; Klein Haneveld et al., 2023; Parsons, 2021; Whalley, 1995). Whereas these patients remain particularly vulnerable, Kellmeyer et al. (2019) suggest that they may actually stand to benefit the most from XR, as they are often confined to secure and sterile environments. While this may be true, it is questioned whether immersion in forensic settings, for instance, infringes the right to mental integrity without valid consent, since such technologies aim to monitor and alter an offender’s mental state and behavior (Ligthart et al., 2022). It is argued that clinicians should therefore practice more diligence when considering the ethical risks of using technologies with such vulnerable patients (Parsons, 2021).

- Theme 3. Data privacy risks and confidentiality concerns

While clinical XR may appear to offer a higher degree of confidentiality (Botella et al., 2004), concerns related to data privacy with implications for non-maleficence persist as ethical concerns: (1) data sensitivity and security; and (2) governance, ownership, and secondary use (Botella et al., 2009; Fromberger et al., 2018; Luxton & Hudlicka, 2021; Marcoux et al., 2021; Marloth et al., 2020; Ozerol & Andic, 2023; Parsons, 2021).

*Data sensitivity and security.* As the technology continue to be developed by private companies, the collected data may be used for other purposes (Marcoux et al., 2021; Marloth et al., 2020; Ozerol & Andic, 2023). XR environments, mainly when associated with wearable sensors, gather a large amount of personal information about the patient, including eye movements, behavioral response patterns, and motor as well as emotional responses, raising concerns of how to guarantee data security (Luxton & Hudlicka, 2021; Marcoux et al., 2021; Marloth et al., 2020; Ozerol & Andic, 2023; Parsons, 2021). It remains questionable to what extent, for instance, a patient’s voice recording will be stored securely, mostly when there are instances of security leaks caused by software (Ozerol & Andic, 2023). In such cases, a patient may reveal issues, thoughts, and emotions that could have adverse repercussions on their life if they were disclosed to others (Luxton & Hudlicka, 2021). This may therefore disturb the principle of confidentiality and bring forth harm to patients (Ozerol & Andic, 2023). Moreover, virtual environments used in non-clinical settings, such as the case of artificial agents, may lead to login details and personal data being shared, thereby threatening personal privacy (Parsons, 2021). Hackers also present a risk, potentially exploiting sensitive data (Luxton & Hudlicka, 2021). Mental health professionals should inform patients of these limitations, as discussed in the section on consent, and ensure patient data is stored and transmitted securely (e.g., password-protected) (Parsons, 2021). Concerning storage, another important privacy-related query is whether data should be made available for data mining to improve algorithms (Marloth et al., 2020).

*Governance, ownership, and secondary use.* As the technology continue to be developed by private companies, collected data may be used for other purposes (Marcoux et al., 2021; Marloth et al., 2020; Ozerol & Andic, 2023). It remains an open question whether, and under what conditions, voice recordings or other data are stored securely, how long they are retained, and whether they become available for data mining to improve algorithms (Marloth et al., 2020). Such practices implicate ownership, consent for secondary use, and cross-context data flows. There is a necessity of regulating and ensuring ethical principles are followed by the widespread use of technology in mental health settings (Marcoux et al., 2021; Ozerol & Andic, 2023). These privacy and confidentiality risks directly engage the principle of non-maleficence, as preventable data harms (e.g., breaches, misuse, unauthorized inferences) must be minimized.

- Theme 4. Clinical liability and regulation

Clinical liability concerns were raised by several authors (*n* = 7) and are is divided into 3 subthemes: (1) autonomous systems, responsibility, and liability; (2) clinician competence and training; and (3) norms, protocols, and regulation (Anderson et al., 2004; Chung et al., 2021; Geraets et al., 2021; Luxton & Hudlicka, 2021; Marcoux et al., 2021; Marloth et al., 2020; Parsons, 2021; Rizzo & Koenig, 2017).

*Autonomous systems, responsibility, and liability.* The use of clinical XR without the oversight of trained professionals, such as in the case of more automated virtual agents, may bring several ethical dilemmas (Marloth et al., 2020). The decision-making capacity of autonomous systems and the allocation of responsibility/liability to ensure patient safety have been questioned (Luxton & Hudlicka, 2021; Marcoux et al., 2021). Examples include virtual agents persuading patients to adopt continuously “healthier” behavior, thereby potentially limiting autonomy (Marcoux et al., 2021), and increases in self-diagnosis/self-treatment with risks of misdiagnosis, mistreatment, and aggravation of existing conditions (Marloth et al., 2020). In scenarios where a patient reveals suicidal or violent thoughts, the responsibility of the agent versus the developer remains unresolved (Luxton & Hudlicka, 2021; Marcoux et al., 2021); many ethical requirements expected of clinicians (e.g., duty-to-warn) have not been fully examined for autonomous characters (Luxton & Hudlicka, 2021). Notably, such agents cannot accept appropriate responsibility nor bear moral consequences (Luxton & Hudlicka, 2021). These issues can arise when XR developments are implemented without sufficient validation or when they omit important patient data (Marcoux et al., 2021; Rizzo & Koenig, 2017).

*Clinician competence and training.* The competency of the therapists has also been put into question, stating that health professionals should be competent in the technical and ethical use of XR (Anderson et al., 2004; Chung et al., 2021; Rizzo & Koenig, 2017). As shown by Chung et al. (2021), clinicians identified knowledge and skills gaps that needed to be addressed to feel confident implementing VR. Training is necessary in technical XR skills, assessing patient suitability and managing ethical and safety risks for professionals to be qualified (Anderson et al., 2004; Chung et al., 2021; Rizzo & Koenig, 2017). Such lack of knowledge could have detrimental effects on patients.

*Norms, protocols, and regulation.* On an ethical basis, providers cannot rely entirely on such technologies due to the lack of adequate norms ensuring safe and ethical use (Parsons, 2021). Harm can occur from inadequate guidelines or monitoring of adverse effects (Chung et al., 2021). Participants in the study by Chung et al. (2021) felt that specific protocols would need to be developed to promote safe and ethical usage. Patient misinformation may also occur in the absence of norms and guidelines (Marcoux et al., 2021).

Authors recommend an ethical use of XR grounded in principles that respect patients’ rights and protect from harm by minimizing potential adverse effects—for example, creating algorithms to reliably indicate its presence during XR and using pre–post measures for simulator-sickness monitoring (Bell et al., 2020; Fromberger et al., 2018; Kellmeyer, 2018; Marloth et al., 2020; Ozerol & Andic, 2023; Parsons, 2021; Selaskowski et al., 2024).

- Theme 5. Fostering inclusiveness and equity in XR development

Other considerations on inclusiveness and equity have been associated with the development of virtual scenarios or characters: (1) Representation, normative assumptions, and bias; and (2) Protection for vulnerable users and governance (Campaner & Costerbosa, 2023; Fromberger et al., 2018; Kellmeyer, 2018; Luxton & Hudlicka, 2021; Marcoux et al., 2021; Marloth et al., 2020).

*Representation, normative assumptions, and bias.* As Marloth et al. (2020) note, program developments require a definition of *normality*, which remains a matter of debate universally, to evaluate and allow following changes in dysfunctional functions. Designers must carefully consider patients’ preferences and the context of use during the development and deployment process (Luxton & Hudlicka, 2021). This is especially important in the case of automated characters and conversational agents that are developed based on knowledge databases, which may be susceptible to bias and may not pay adequate attention to cultural nuances (Luxton & Hudlicka, 2021; Marcoux et al., 2021). In a co-design study in forensic youth care, stakeholders (experiential experts and professionals) emphasized that “reality equals diversity,” arguing that diversity must be clearly visible in content (Klein Schaarsberg et al., 2024). Such bias can introduce systematic errors leading to inequalities in diagnosis and treatment (Marcoux et al., 2021), engaging justice (fairness) and non-maleficence (avoidance of preventable harm).

*Protections for vulnerable users and governance.* The creation of these entities leads to ethical considerations concerning their degree of identity (Campaner & Costerbosa, 2023). When creating personalized virtual characters for patients, clinicians should support patients with vulnerabilities, such as impaired body image, to prevent adverse effects on body image (e.g., eating disorders) (Kellmeyer, 2018). For scenarios involving forensic populations (e.g., diagnosis or treatment of child abusers), developers should not design sexually explicit content nor record real-life children (Fromberger et al., 2018). Actors used for motion capture should likewise be informed of the context in which the data will be used (Fromberger et al., 2018). Finally, albeit more marginally, some authors caution against the potential political misuse of extended reality as a tool for control and normalization toward certain political ideals, highlighting the importance of promoting independent regulation. These safeguards operationalize non-maleficence (risk minimization) while promoting justice (equitable protection across groups).

## 4. Discussion

This review examined the ethical concerns regarding the applications of XR in mental health care. We synthesize concerns across five themes: i) Balancing beneficence and non-maleficence as a question of patient safety, (ii) Altering autonomy by altering reality and information, (iii) data privacy risks and confidentiality concerns, (iv) clinical liability and regulation, and v) fostering inclusiveness and equity in XR development.

Most of the reviewed studies emphasized the balance between beneficence and non-maleficence, with some addressing autonomy, while the principle of justice—the fourth ethical principle in medical bioethics (T. Beauchamp & Childress, 2019)—was notably underexplored. Yet, no single ethical principle should outweigh another, underscoring the need for clinical judgment and the contextualization of XR use based on individual patient needs—an area scarcely addressed in the existing literature on XR in psychiatric settings. A likely reason is that most studies prioritize immediate, patient-level safety and feasibility, whereas justice (fair access, representation, distribution of benefits/burdens) requires system-level data and equity metrics rarely collected. Moreover, the corpus is dominated by non-empirical work from early-adopter services with limited stakeholder diversity, sparse reporting of sociodemographics, and little co-design, making equity effects less visible. As XR deployment broadens, embedding equity indicators, participatory methods, and transparent reporting on inclusion will be needed to make justice analysable alongside the other principles.

Most authors stated ethical concerns about the use of XR primarily for patient safety and effects of simulating reality. There is a clear need to monitor and ensure patient safety during and after the use of XR. Furthermore, the prefabricated realities may cause patient deception and lead to negative effects (e.g., attachment to the virtual agent, addiction, manipulation, altered therapeutic relationship) as well as developmental biases. Unfortunately, long-term effects remain insufficiently documented for both XR applications and embodied virtual agents (Rudschies & Schneider, 2024). This evidentiary gap raises specific ethical concerns: when longer-term benefits and harms are unknown, patients cannot be fully informed about material risks and alternatives, which challenges the validity of informed consent and the respect for autonomy.

Although such risks exist, many studies, including meta-analyses have shown acceptability and benefits of using XR applications in psychiatry, even in patients with severe mental disorders, such as schizophrenia (e.g., (Carl et al., 2019; Dellazizzo et al., 2020; Freeman et al., 2017; Maples-Keller et al., 2017; Rus-Calafell et al., 2018; Valmaggia et al., 2016)). While genuine human–human interactions may appear preferable to human–machine contact and lead to better therapeutic alliance, mostly in vulnerable patients, evidence remains limited (Kellmeyer et al., 2019; Rudschies & Schneider, 2024; Selaskowski et al., 2024). XR for clinical use, for instance, may seem like a non-invasive technique in comparison to pharmacological or other medical techniques (Hardy et al., 2019). Hence, as stated by Kellmeyer et al. (2019), professionals have an ethical responsibility to ensure patients benefit from new technologies and consider patient vulnerability that may compromise safety. This could, however, be hindered by the lack of competencies and knowledge in XR use from professionals (Chung et al., 2023), which may bring forth additional questions concerning responsibility and liability (Luxton & Hudlicka, 2021; Marcoux et al., 2021; Marloth et al., 2020). In these newer automated developments and research area, it is thus crucial to rapidly involve patients, technical and mental health professionals (Brett et al., 2014).

The use of XR and artificial agents in mental health care introduces important ethical complexities, particularly when simulated environments alter patients’ perceptions of reality and influence the development of a Digital Therapeutic Alliance (DTA). Multiple studies from this review have explored how immersive technologies may fabricate or distort reality, raising concerns about deception, loss of authenticity, and compromised autonomy. In these environments, patients may struggle to discern whether they are interacting with a human clinician or an autonomous system, which undermines informed consent and the authenticity of the therapeutic bond (Luxton & Hudlicka, 2021; Marcoux et al., 2021). When patients cannot tell who (or what) is delivering care—and the degree of autonomy, human oversight, and data practices involved—their ability to receive and understand material information about risks, alternatives, and the right to refuse is limited, thereby compromising the essential elements of valid informed consent and diminishing the authenticity of the therapeutic bond. Furthermore, patients may develop emotional attachments to avatars or agents that simulate empathy and responsiveness, leading to artificial relationships that lack mutuality and reciprocity, which are key components of a traditional therapeutic alliance (Luxton & Hudlicka, 2021; Selaskowski et al., 2024).

These dynamics challenge the integrity of the DTA, which relies on shared goals, task agreement, and a genuine therapeutic bond (Malouin-Lachance et al., 2025; Beatty et al., 2022; Goldberg et al., 2022). When patients are unaware of the artificial nature of the agent or the environment, the alliance may be built on a foundation of illusion rather than trust. Campaner and Costerbosa (2023) argue that a certain degree of deception is inherent in avatar-based therapies, where patients engage in dialogue with fictional entities. While this imaginative engagement may enhance insight and therapeutic efficacy, it also risks undermining the patient’s autonomy and capacity for informed decision-making if the artificiality is not fully disclosed. Moreover, immersive XR environments may impose prefabricated constraints or behavioral nudges that subtly manipulate patients without their awareness, further complicating the ethical landscape of the DTA (Ligthart et al., 2022; Marcoux et al., 2021). To preserve the ethical integrity of the DTA, clinicians and developers must ensure transparency regarding the agent’s autonomy, establish clear boundaries in therapeutic dialogue, and adhere to deontological guidelines that protect against undue influence or coercion (Malouin-Lachance et al., 2025; Beatty et al., 2022; Ligthart et al., 2022; Goldberg et al., 2022).

Patients, regardless of their capacity to consent, therefore have the right to be informed about the different features concerning the use of XR (e.g., nature of XR, limitations, unpleasant effects, data collection and security). Applications should be used transparently and respect patients’ autonomy as well as confidentiality. While XR technologies offer immersive and interactive therapeutic environments, the question arises as to what extent these features should be revealed to patients. Debate persists about the appropriate level of disclosure for technical/immersive features much like how certain aspects of psychotherapy are not fully disclosed to maintain therapeutic efficacy (Barrett & Berman, 2001). On one hand, comprehensive disclosure aligns with ethical principles of autonomy and informed consent. On the other hand, withholding some information—such as immersive elements or data processing details—could be justified if deemed beneficial to the therapeutic process. Thus, developing clear guidelines on the degree of disclosure remains crucial to balancing patient autonomy with clinical efficacy, particularly in psychiatric contexts where patients may be more vulnerable to XR-induced distortions or misunderstandings.

The involvement of various stakeholders is crucial to maintaining an ethical perspective in the development and implementation of these emerging mental healthcare technologies. Currently, these increasingly sophisticated technologies are becoming pervasive in everyday life, leaving little time to pause and reassess ethical frameworks to effectively guide their use in clinical practice. While the principles of biomedical ethics remain foundational (T. Beauchamp & Childress, 2019), complementary frameworks have emerged such as the *Montreal Declaration for a Responsible Development of Artificial Intelligence* (Montréal Declaration, 2018) and the World Health Organization (WHO) (2021) which outline principles to guide digital and AI-related practices. They articulate well-being, safety, public interest, autonomy, responsibility, accountability, and the patient–machine relationship. However, these remain high-level and can fall short of clinicians’ needs for actionable protocols (Chung et al., 2021). Participatory, value-based development work in forensic mental health shows how end-users and professionals can co-create design requirements and iteratively test prototypes, translating stakeholder input into concrete design and reporting decisions (Kip et al., 2019). Similarly, implementation research indicates that introducing VR into clinical services depends on early training, organizational fit, and explicit, co-designed implementation strategies that go beyond efficacy testing (M. T. E. Kouijzer et al., 2024). Together, these approaches help operationalize ethical principles into practice-ready guidance. Building on the Montreal Declaration, Laverdière and Régis (2023) recently published a report aimed at supporting the establishment of a prototype code of ethics for AI use in health and human relations. However, they emphasize that ethical practice requires more than mere adherence to a code—it necessitates critical thinking and ethical reasoning from users, as Madary and Metzinger (2016) previously argued. In this context, the focus should shift toward developing guidelines that not only regulate but also personalize practices, rather than imposing rigid norms (Laverdière & Régis, 2023). Consequently, integrating diverse stakeholders, including citizens from various backgrounds, becomes essential for reflecting on the development of these cutting-edge AI technologies and for co-constructing practical guidelines that will govern their use in clinical settings (World Health Organization (WHO), 2022).

## 5. Limitations

This study presents certain limitations. Scoping reviews typically do not assess the quality of included studies, potentially integrating research with varying methodological rigor (Peters et al., 2020). This can be particularly challenging in emerging fields like XR, where empirical data are limited, and much of the literature consists of narratives reviews or commentary or opinion papers rather than robust empirical studies. This context can foster an echo chamber effect, similar to that observed in social media networks, where the same articles are repeatedly discussed, amplifying their perceived influence and reinforcing their reach. Regarding our analysis process, codes and subcodes were developed iteratively by two reviewers with documented consensus meetings; we did not compute a formal inter-rater reliability coefficient, which enhances reflexivity but may limit reproducibility. Furthermore, Conceptual heterogeneity across included studies (e.g., varying definitions of XR/VR/AR) and our choice not to require “sense of presence” as an inclusion criterion may introduce classification variability.

## 6. Conclusions

To conclude, this review highlighted several ethical concerns following the use of XR technologies in the domain of mental health care. Despite the limited number of studies reviewed, many of the identified concerns—particularly those related to more automated technologies—are relevant across other healthcare domains and emerging technologies, such as AI applications. Because scoping reviews do not appraise study quality and the evidence base is largely non-empirical, the claims advanced here should be read as a map of ethical salience. There is a pressing need for more transdisciplinary research with a bioethical focus as clinical care increasingly integrates novel technologies.

For researchers, priorities include prospective and longitudinal studies, mixed-methods evaluations in real-world services, equity and inclusion metrics, and implementation reporting that makes ethical trade-offs explicit. For clinicians, a precautionary approach is warranted now: ensure competence and training, obtain and document robust informed consent, monitor and mitigate adverse effects, and apply strong data-governance safeguards while tailoring use to patient context.

Active participation from all stakeholders, including patients, researchers, clinicians, and XR developers, is crucial in shaping the ethical use of XR through its development, implementation, and evaluation. Evidence-based guidelines are essential to ensure safe and ethically sound practices; however, they cannot replace the clinical judgment necessary to tailor interventions to the specific context of each patient. These guidelines may also be a framework for newer technological developments, notably with the blooming use of artificial intelligence and their associated algorithms in mental health. Ultimately, significant research gaps persist, particularly regarding XR and the development of a comprehensive regulatory framework that ensures responsible and patient-centered implementation.

## Figures and Tables

**Table 1:** Population, concept and context.

Population	The target population includes all individuals who may be involved with the concept, namely individuals living with a mental health problem, their relatives, healthcare professionals, policymakers, and XR system developers.
Concept:	Ethical dilemmas in the use of XR (virtual reality, avatar, virtual agents, computer-simulated)
Context	Clinical mental health and psychiatry

**Table 2:** Summary of articles included in the scoping review.

Authors-Year (Country)	Discipline	Study Design	Aim	Sample Size Target Population	Type of Technology	Ethically Related Themes
(Anderson et al., 2004) (USA)	Psychology	Narrative review; no specific methodology was provided	To review the empirical literature pertaining to the use of computer-supported cognitive-behavioral treatment of anxiety disorders	N/A Anxiety Disorders	Computer-supported cognitive behavioral treatment, including VR	Exacerbation of symptoms related to the use of technology Competence of clinicians Compromised therapist–client relationship Conflict of interest
(Bell et al., 2020) (Australia and UK)	Psychiatry, Psychology & Neuroscience	Narrative review; no specific methodology was provided	To provide a summary of the advantages of using VR for assessment in mental health	N/A Mental Health Disorders	VR	Privacy, Confidentiality, Transparency, Security, Ownership of data
(Botella et al., 2009) (Spain)	Psychology	Opinion	To explore the ethical issues encountered by the authors in the course of their research and clinical practice	N/A Mental Health Disorders	VR, Augmented reality, Telepsychology, Ubiquitous computing, and Persuasive computing	Framework and contexts of intervention Confidentiality, Safety, Cybersickness, Exacerbation of symptoms related to the use of technology
(Campaner & Costerbosa, 2023) (Italy)	Philosophy & Psychiatry	Book chapter	By discussing selected epistemological and ethical aspects, this chapter aims to offer a tentative evaluation of the trade-offs between the promises and limitations of Avatar therapy	N/A Psychotic Disorders	VR (Avatar Therapy)	Framework and contexts of intervention, Informed consent, Patient autonomy, Compromised therapist–client relationship
(Chivilgina et al., 2021) (Switzerland)	Biomedical Ethics	Descriptive review	To systematize ethical concerns related to digital technologies in mental health, with a particular focus on individuals suffering from schizophrenia	Full-text articles (*n* = 264) Schizophrenia spectrum disorders	Digital technologies, including VR	Lack of evidence on efficacy and impact on self-perception, Lack of clear standards for safety, Unclear ownership data, Lack of user-centered design, Unclear roles and boundaries of technology in therapy
(Chung et al., 2023) (Australia)	Psychology & Psychiatry	Qualitative design	(1) synthesis perspectives of staff working in private mental healthcare and (2) use the Theoretical Domains Framework and Behaviour Change Wheel to identify mechanisms of change targets and intervention functions to facilitate its clinical implementation	Clinicians (*n* = 14), service managers (*n* = 5) Private mental health hospital	VR	Exacerbation of symptoms related to the use of technology, Cybersickness, Knowledge and skills gaps among clinicians
(Chung et al., 2021) (Australia)	Psychology & Psychiatry	Qualitative design	This study aimed to explore the perspectives of staff working in the private mental health sector around the use of therapeutic VR, including potential implementation barriers and facilitators	Clinicians (*n* = 14), service managers (*n* = 5) Private mental health hospital	VR	Resourcing constraints (i.e., clinical guidelines, training programs), Safety and ethical concerns, Negative staff attitudes towards technology, VR system limitations, Knowledge and skills gaps among clinicians
(Cornet & Van Gelder, 2020) (The Netherlands)	Psychology	Review; no specific methodology was provided	To explore the possibilities for VR application within criminal justice practice	N/A Forensic and criminal justice	VR	Exacerbation of symptoms related to the use of technology, Resourcing constraints, Privacy, Ownership of data, Risk of Misuse
(Fromberger et al., 2018) (Germany)	Psychiatry	Review; no specific methodology was provided	To examine current and potential uses of VR in forensic psychology, focusing on risk assessment, treatment, and behavioral training in forensic mental health	N/A Forensic mental health (child abuse)	VR	Exacerbation of symptoms related to the use of technology, Ownership of data, Confidentiality, Transparency, Risk of Misuse
(Georgieva & Georgiev, 2019) (Bulgaria)	Philosophy	Theoretical study based on the philosophical idea of the narrative self	To study the role of VR as a tool for the creation of stories with the concept of the self as a narrator and the life of the self as a storyline	N/A	VR	Exacerbation of symptoms related to the use of technology, Safety concerns, Resourcing constraints, Complications in distinguishing between ‘real’ and virtual experiences, Risk of addiction or escapism due to the immersive appeal of simulated alternative realities
(Geraets et al., 2021) (The Netherlands)	Psychiatry	Review; no specific methodology was provided	To review current advances in immersive VR-based therapies for mental disorders	N/A	VR	Resourcing constraints (i.e., clinical guidelines, training programs), Professional guidelines are needed to promote its safe and ethical use
(Kellmeyer, 2018) (Germany)	Neuroethics, Neurophilosophy, Neurology & Psychiatry	Commentary	To review clinical uses of VR in neurology and psychiatry, introduces key concepts from neurophilosophy and VR research, and highlights ethical concerns and adverse effects of immersive VR in therapy	N/A Neurology, psychiatry, and many other medical fields	Standalone and hybrid VR systems	“A user centered approach that is informed by the target patients’ needs and capabilities could help to build beneficial systems for VR therapy.” Effects of VR on vulnerable patients, Transparency, Exacerbation of symptoms related to the use of technology, Cybersickness
(Kellmeyer et al., 2019) (Germany)	Biomedical Ethics	Commentary	To analyze and highlight the ethical issues related to the use of VR systems in the treatment of vulnerable patients	N/A Neurology, psychiatry, and clinical psychology	VR	Informed consent, Patient autonomy, Compromised therapist–client relationship, Complications in distinguishing between ‘real’ and virtual experiences
(Klein Haneveld et al., 2023) (The Netherlands)	Psychology	Exploratory qualitative	To identify if, how and for whom DEEP can be of added value in forensic mental healthcare	Healthcare providers (*n* = 24), forensic psychiatric inpatients (*n* = 13)	VR	Exacerbation of symptoms related to the use of technology, Informed consent of patients. Both patients and clinicians should be involved from the outset in the evaluation and implementation processes. A multilevel approach should guide the development of implementation strategies.
(Klein Schaarsberg et al., 2024) (The Netherlands)	Psychiatry	Qualitative	By presenting a detailed example of their VR developmental process, specifically focusing on ethnic representation in this virtual environment and related ethical aspects, the authors aim to positively contribute to the creation of ethically sound therapeutic VR-applications	Adolescents with Disruptive Behavior Problems (*n* = 10), Experiential Expert (*n* = 4), Youth care Professional (*n* = 4)	VR	Ethical considerations regarding ethnic representation within virtual environments
(M. T. E. Kouijzer et al., 2024) (The Netherlands)	Psychology	Qualitative	To gain insight from the impressions of both patients and healthcare providers concerning the integration of VR in practice	Healthcare providers (*n* = 10), forensic psychiatric outpatients (*n* = 8)	VR	Virtual trauma, Risk of re-traumatization through simulated painful experiences. “The initial step of integrating VR into practice demands careful planning and a personalized approach. These efforts are crucial to fully realize its potential in clinical practice.”
(Kruk et al., 2019) (Poland)	Psychiatry	Commentary	To discuss the basic concepts of the VR environment and its impact on the users	N/A Mental health disorders	VR	Possible incomplete representation of reality, Complexity of the problem of VR reception by the mind generating its own “VR”, Exacerbation of symptoms related to the use of technology
(Ligthart et al., 2022) (The Netherlands)	Bioethics, Technology, Law, Criminality & Mental Health	Commentary The authors examine two normative frameworks: human rights and ethical principlism	To broaden the current ethical and legal debate on Extended Reality applications to their use in the resocialization of criminal offenders, mainly focusing on forensic patients	Criminal justice and Forensic mental health	VR	“Offering XR in forensic treatment and resocialization should be approached with caution, since it could potentially infringe fundamental rights over the mind, increase the users’ vulnerability and dependency, stigmatize and adversely affect their authenticity and moral agency.”
(Luxton & Hudlicka, 2021) (USA)	Psychiatry	Book chapter	To provide an overview of embodied intelligent virtual agents (IVAs) and non-embodied conversational agents, such as chatbots, highlighting their applications in behavioral and mental healthcare. The focus is on their roles in training, coaching, behavioral modeling, and delivering limited treatment functions, particularly in highly scripted, protocol-based interventions	N/A Mental healthcare	Embodied intelligent virtual agents (IVAs) and nonembodied conversational agents (CAs)	Potential concerns include cultural, demographic, or linguistic implicit biases introduced during development, as well as a lack of transparency regarding the agent’s behavioral choices
(Marcoux et al., 2021) (Canada)	Psychology	Narrative literature review	To inform relevant actors, including clinicians, on the potential of virtual characters in mental healthcare practices and to raise awareness on societal challenges regarding their use	N/A Mental healthcare	Digitally represented virtual characters	Socioeconomic and cultural inequalities, Cybersickness, Resourcing constraints, Privacy, Confidentiality, Transparency, Security, Ownership of data, Person-centered approach
(Marloth et al., 2020) (Germany)	Psychiatry	Review	To address the different themes and recommend the development of an ethical framework for the clinical use of VR	N/A Mental health disorders	VR	Reality and its representation, Virtual trauma, Patient autonomy, Privacy, Self-Diagnosis and Self-Treatment, Expectation bias
(Ozerol & Andic, 2023) (Turkey)	Psychology	Review; no specific methodology was provided	To explore the past, present, and future of schizophrenia treatment, including effective interventions, VR therapy, the fundamentals of avatar therapy, studies on its effectiveness, and related ethical considerations	N/A Schizophrenia spectrum disorders	VR (Avatar Therapy)	Confidentiality, Privacy, Patient’s sense of security, “It is not clear how the therapeutic relationship with the therapist will be affected”, Risk of Misuse
(Parsons, 2021) (USA)	Computational Neuropsychology	Review; no specific methodology was provided	To discuss some of the ethical issues involved in the clinical use of novel technologies	N/A Mental health disorders	Virtual environment technologies	Privacy, Security, Ownership of data, Free and informed consent, Patient autonomy, Risk of Misuse
(Pellicano, 2023) (Germany)	Psychology	Review; no specific methodology was provided	To explain efficacy of VR exposure therapy in treating post-traumatic stress disorder	N/A Post-traumatic stress disorder	VR exposure therapy (VRET)	Resourcing constraints, Risk of re-traumatization through simulated painful experiences, Need for patient-centered design
(Rizzo & Koenig, 2017) (USA and Australia)	Psychology	Review; no specific methodology was provided	To give an overview of VR technology, its use in clinical settings over the past 20 years, and the main benefits it offers for therapy. It also looks at how ready VR is for clinical use, covering its scientific foundation, existing research, practical issues like cost and usability, and important ethical concerns	N/A Clinical health conditions, including mental health	VR	Clinicians’ boundaries of competence: Professional guidelines are needed to promote its safe and ethical use, Self-Diagnosis and Self-Treatment
(Selaskowski et al., 2024) (Germany)	Psychiatry and Psychotherapy	Commentary	To discuss the unique issues associated with the incorporation of VR in clinical research	N/A Mental healthcare	VR	Safety, Cybersickness, Exacerbation of symptoms related to the use of technology, Compromised therapist–client relationship
(Veling et al., 2021) (The Netherlands)	Psychiatry	Quantitative	To investigate the immediate effects of VR relaxation on negative and positive affective states and short-term effects on perceived stress and symptoms in patients with a psychiatric disorder, compared to standard relaxation exercises.	Patients with Mental Health Disorders (*n* = 50)	VR	Cybersickness, Exacerbation of symptoms related to the use of technology
(Washburn et al., 2021) (USA)	Social Work	Review; no specific methodology was provided	To provide an overview of older adult substance misuse and treatment needs, followed by a review of ethics and general safety related considerations for using VR based intervention approaches with an older adult population	N/A Older adults impacted by substance misuse	VR	Exacerbation of symptoms related to the use of technology, Complications in distinguishing between ‘real’ and virtual experiences
(Whalley, 1995) (UK)	Psychiatry	Review; no specific methodology was provided	To explore the ethical implications of using VR in medical research and patient care, particularly focusing on the risks and challenges of introducing vulnerable or mentally impaired patients to VR environments	N/A Clinical care, including mental health	VR	Exacerbation of symptoms related to the use of technology, Informed consent, Patient autonomy

N/A: Not applicable.

## Data Availability

No new data were created or analyzed in this study. Data sharing is not applicable to this article.

## Supplementary Materials

The following supporting information can be downloaded at: https://www.mdpi.com/article/10.3390/bs15101431/s1. Reference Tricco et al. (2018) is cited in the supplementary materials.
