# Supplementary material for: What Are the Ethical Issues Surrounding Extended Reality in Mental Health? A Scoping Review of the Different Perspectives
Source: Behav Sci (Basel). 2025 Oct 21;15(10):1431. doi: 10.3390/bs15101431 (PMC12561320; doi:10.3390/bs15101431)
Supplement: Supplementary file 1 [file behavsci-15-01431-s001.zip › Supplementary material 2_search strategy.pdf]

Supplementary material 2

Table S2. Search strategy

Ethical Issues	Virtual Reality/Artificial intelligence/Avatars	Psychiatry/Mental Disorders
**Descriptors (Mesh)**<br><br>"Ethics"[Mesh]<br>"ethics" [Subheading]<br>"Principle-Based Ethics"[Mesh]<br>"Morals"[Mesh:NoExp]<br>"Philosophy"[Mesh:NoExp]<br>"Personal Autonomy"[Mesh:NoExp]<br>"Psychological Well-Being"[Mesh]<br>"Beneficence"[Mesh]<br>"Confidentiality"[Mesh:NoExp]<br>"Informed Consent"[Mesh]<br>"Privacy"[Mesh]<br>"Health Equity"[Mesh]<br>"Diversity, Equity, Inclusion"[Mesh]<br>"Health Services Accessibility"[Mesh]<br>"Professional-Patient Relations"[Mesh]<br>"Social Responsibility"[Mesh]<br>"Safety"[Mesh]<br>"Psychological Safety"[Mesh]<br>"Social Cohesion"[Mesh]<br>"Social Values"[Mesh]<br><br>**Keywords (title/abstract)**<br><br>Ethical<br>Ethic(s)<br>Ethically<br>Bioethics(s)<br>Bioethical	**Descriptors (Mesh)**<br><br>"Artificial Intelligence"[Mesh:NoExp]<br>"Virtual Reality"[Mesh]<br>"Virtual Reality Exposure Therapy"[Mesh]<br><br>**Keywords (title/abstract)**<br><br>Virtual reality(ies)<br>Avatar(s)<br>Artificial intelligence<br>Augmented reality(ies)<br>Virtual simulation(s)<br>Simulated environment(s)<br>Virtual agent(s)<br>VR<br>VRET<br>AI<br>Computer-simulated<br>Computer-generated<br>Immersive digital<br>Synthetic reality(ies)	**Descriptors (Mesh)**<br><br>"Mental Disorders"[Mesh:NoExp]<br>"Anxiety Disorders"[Mesh]<br>"Mood Disorders"[Mesh]<br>"Personality Disorders"[Mesh]<br>"Schizophrenia Spectrum and Other Psychotic Disorders"[Mesh]<br>"Trauma and Stressor Related Disorders"[Mesh]<br>"Autism Spectrum Disorder"[Mesh]<br>"Attention Deficit Disorder with Hyperactivity"[Mesh]<br>"Substance-Related Disorders"[Mesh]<br>"Neurocognitive Disorders"[Mesh:NoExp]<br>"Dementia"[Mesh]<br>"Mental Health"[Mesh]<br>"Psychiatry"[Mesh]<br>"Mental Health Services"[Mesh]<br>"Vulnerable Populations"[Mesh]<br><br>**Keywords (title/abstract)**<br><br>Psychiatric<br>Psychiatry<br>Mental disorder(s)<br>Mental illness(es)

bioethically<br>Neuroethical<br>Neuroethic(s)<br>neuroethically<br>Moral(s)<br>Philosophy<br>Philosophical<br>Neurophilosophy<br>Neurophilosophical<br>Autonomy<br>Self-determination<br>Well-being<br>Wellbeing<br>Wellness<br>Beneficence<br>Non-maltreatment<br>confidentiality<br>consent<br>consequentialism<br>deontology(ical)<br>equity(ies)<br>inclusion(sive) AND diversity<br>ethos<br>humility<br>integrity<br><br>accessibility<br>access<br>AND<br>Service(s)<br>Care<br>Healthcare<br><br>explicability<br>caution<br>patient ADJ relationship(s)<br>responsibility(ies)<br>safety<br>safe<br>security<br>secure<br>cohesion<br>solidarity(ies)<br>transparency(ies)<br>utilitarianism<br>value(s)<br>privacy(ies)		Mental health<br>Mental healthcare<br>Schizophrenia(ic)<br>Psychosis<br>Psychotic<br>Hallucination(s)<br>Anxiety disorder(s)<br>Phobia(s)<br>panic<br>Post-traumatic<br>Posttraumatic<br>PTSD<br>Depression<br>Depressive<br>Bipolar disorder(s)<br>Mood disorder(s)<br>Affective disorder(s)<br>Personality disorder(s)<br>Autism<br>Autistic<br>ASD<br>Attention deficit hyperactivity disorder(s)<br>Adhd<br>Substance related disorder(s)<br>Substance use(s)<br>Substance misuse(s)<br>Substance abuse(s)<br>Drug(s) related disorder(s)<br>Drug(s) use(s)<br>Drug(s) misuse(s)<br>Drug(s) abuse(s)<br>Addiction(s)<br>Nicotine<br>Tobacco<br>Cannabis<br>Marijuana<br>Alcohol(ism)<br>Cocaine<br>Neurocognitive disorder(s)<br>Dementia(s)<br>Alzheimer<br>Vulnerable population(s)

private life		

Concept 1

"Ethics"[Mesh] OR "ethics" [Subheading] OR "Principle-Based Ethics"[Mesh] OR "Morals"[Mesh:NoExp] OR "Philosophy"[Mesh:NoExp] OR "Personal Autonomy"[Mesh:NoExp] OR "Psychological Well-Being"[Mesh] OR "Beneficence"[Mesh] OR "Confidentiality"[Mesh:NoExp] OR "Informed Consent"[Mesh] OR "Privacy"[Mesh] OR "Health Equity"[Mesh] OR "Diversity, Equity, Inclusion"[Mesh] OR "Health Services Accessibility"[Mesh] OR "Professional-Patient Relations"[Mesh] OR "Social Responsibility"[Mesh] OR "Safety"[Mesh] OR "Psychological Safety"[Mesh] OR "Social Cohesion"[Mesh] OR "Social Values"[Mesh] OR Ethic*[TIAB] OR Bioethic*[TIAB] OR Neuroethic*[TIAB] OR Moral*[TIAB] OR Philosoph*[TIAB] OR Neurophilosoph*[TIAB] OR Autonomy[TIAB] OR "Self-determination"[TIAB] OR "Well-being"[TIAB] OR Wellbeing[TIAB] OR Wellness[TIAB] OR Beneficence[TIAB] OR "Non-maltreatment"[TIAB] OR confidentiality[TIAB] OR consent[TIAB] OR consequentialism[TIAB] OR deontolog*[TIAB] OR equit*[TIAB] OR (diversity[TIAB] AND (inclusion[TIAB] OR inclusive[TIAB])) OR ethos[TIAB] OR humility[TIAB] OR integrity[TIAB] OR ((access*[TIAB] OR explicability[TIAB]) AND (service*[TIAB] OR care[TIAB] OR healthcare[TIAB])) OR explicability[TIAB] OR caution[TIAB] OR "patient relationship"[TIAB:~2] OR "patient relationships"[TIAB:~2] OR responsibilit*[TIAB] OR safety[TIAB] OR safe[TIAB] OR security[TIAB] OR secure[TIAB] OR cohesion[TIAB] OR solidarity[TIAB] OR solidarities[TIAB] OR transparency[TIAB] OR transparencies[TIAB] OR utilitarianism[TIAB] OR value*[TIAB] OR privacy[TIAB] OR privacies[TIAB] OR "private life"[TIAB]

Concept 2

"Artificial Intelligence"[Mesh:NoExp] OR "Virtual Reality"[Mesh] OR "Virtual Reality Exposure Therapy"[Mesh] OR "Virtual realit*"[TIAB] OR Avatar*[TIAB] OR "Artificial intelligence"[TIAB] OR "Augmented realit*"[TIAB] OR "Virtual simulation*"[TIAB] OR "Simulated environment*"[TIAB] OR "Virtual agent*"[TIAB] OR VR[TIAB] OR AI[TIAB] OR "Computer-simulated"[TIAB] OR "Computer-generated"[TIAB] OR "Immersive digital"[TIAB] OR "Synthetic realit*"[TIAB]

Concept 3

"Mental Disorders"[Mesh:NoExp] OR "Anxiety Disorders"[Mesh] OR "Mood Disorders"[Mesh] OR "Personality Disorders"[Mesh] OR "Schizophrenia Spectrum and Other Psychotic Disorders"[Mesh] OR "Trauma and Stressor Related Disorders"[Mesh] OR "Autism Spectrum Disorder"[Mesh] OR "Attention Deficit Disorder with Hyperactivity"[Mesh] OR "Substance-

Related Disorders"[Mesh] OR "Neurocognitive Disorders"[Mesh:NoExp] OR "Dementia"[Mesh] OR "Mental Health"[Mesh] OR "Psychiatry"[Mesh] OR "Mental Health Services"[Mesh] OR "Vulnerable Populations"[Mesh] OR Psychiatric[TIAB] OR Psychiatry[TIAB] OR "Mental disorder*"[TIAB] OR "Mental illness*"[TIAB] OR "Mental health"[TIAB] OR "Mental healthcare"[TIAB] OR Schizo*[TIAB] OR Psychosis[TIAB] OR Psychotic[TIAB] OR Hallucination*[TIAB] OR "anxiety disorder*"[TIAB] OR Phobia*[TIAB] OR panic[TIAB] OR "Post-traumatic"[TIAB] OR Posttraumatic[TIAB] OR PTSD[TIAB] OR Depression[TIAB] OR Depressive[TIAB] OR "Bipolar disorder"[TIAB:~2] OR "bipolar disorders"[TIAB:~2] OR "Mood disorder*"[TIAB] OR "Affective disorder*"[TIAB] OR "Personality disorder*"[TIAB] OR Autism[TIAB] OR Autistic[TIAB] OR ASD[TIAB] OR "Attention deficit hyperactivity"[TIAB:~2] OR adhd[TIAB] OR "Substance related disorder*"[TIAB] OR "Substance use*"[TIAB] OR "Substance misuse*"[TIAB] OR "Substance abuse*"[TIAB] OR "Drug* related disorder*"[TIAB] OR "Drug* use*"[TIAB] OR "Drug* misuse*"[TIAB] OR "Drug* abuse*"[TIAB] OR Addiction*[TIAB] OR Nicotine[TIAB] OR Tobacco[TIAB] OR Cannabis[TIAB] OR Marijuana[TIAB] OR Alcohol*[TIAB] OR Cocaine[TIAB] OR "Neurocognitive disorder*"[TIAB] OR Dementia*[TIAB] OR Alzheimer[TIAB] OR "Vulnerable population*"[TIAB]

Free-text vocabulary search – other databases

Ethic* OR Bioethic* OR Neuroethic* OR Moral* OR Philosoph* OR Neurophilosoph* OR Autonomy OR "Self-determination" OR "Well-being" OR Wellbeing OR Wellness OR Beneficence OR "Non-maltreatment" OR confidentiality OR consent OR consequentialism OR deontolog* OR equit* OR (diversity AND (inclusion OR inclusive)) OR ethos OR humility OR integrity OR (access* AND (service* OR care OR healthcare)) OR explicability OR caution OR (patient ADJ2 relationship*) OR responsibilit* OR safety OR safe OR security OR secure OR cohesion OR solidarity OR solidarities OR transparency OR transparencies OR utilitarianism OR value* OR privacy OR privacies OR "private life"

"Virtual realit*" OR Avatar* OR "Artificial intelligence" OR "Augmented realit*" OR "Virtual simulation*" OR "Simulated environment*" OR "Virtual agent*" OR VR OR AI OR "Computer-simulated" OR "Computer-generated" OR "Immersive digital" OR "Synthetic realit*"

Psychiatric OR Psychiatry OR "Mental disorder*" OR "Mental illness*" OR "Mental health" OR "Mental healthcare" OR Schizo* OR Psychosis OR Psychotic OR Hallucination* OR "anxiety disorder*" OR Phobia* OR panic OR "Post-traumatic" OR Posttraumatic OR PTSD OR Depression OR Depressive OR (Bipolar ADJ2 disorder*) OR "Mood disorder*" OR "Affective disorder*" OR "Personality disorder*" OR Autism OR Autistic OR ASD OR ("Attention deficit" ADJ2

hyperactivity) OR adhd OR ((substance OR drug*) ADJ2 (disorder* OR "use*" OR misuse* OR abuse*)) OR Addiction* OR Nicotine OR Tobacco OR Cannabis OR Marijuana OR Alcohol* OR Cocaine OR "Neurocognitive disorder*" OR Dementia* OR Alzheimer OR "Vulnerable population*"
